# Exploration of natural products for the development of promising cholinesterase inhibitors in Alzheimer's disease treatment

**DOI:** 10.1016/j.heliyon.2025.e42479

**Published:** 2025-02-05

**Authors:** Hassan Nour, Oussama Abchir, Nouh Mounadi, Abdelouahid Samadi, Belaidi Salah, Samir Chtita

**Affiliations:** a Laboratory of Analytical and Molecular Chemistry, Faculty of Sciences Ben M'Sik, Hassan II University of Casablanca, Casablanca, 7955, Morocco; b Department of Chemistry, College of Science, UAEU, P.O. Box No. 15551, Al Ain, United Arab Emirates; c Group of Computational and Medicinal Chemistry, LMCE Laboratory, University of Biskra, BP 145, Biskra, 707000, Algeria

**Keywords:** Alzheimer's disease, Molecular docking, Medicinal plants, Cholinesterase inhibitors, Molecular dynamics, ADMET

## Abstract

Cholinesterase enzymes (BuChE and AChE) are privileged biological targets for the symptomatic treatment of Alzheimer's disease. Indeed, inhibition of cholinesterase enzymes has been proven to improve the neurotransmission mechanisms in Alzheimer's disease patients. In this investigation, we attempt to highlight new cholinesterase inhibitors from natural products. For this purpose, secondary metabolites (299 phytoconstituents) of twenty-eight Medicinal plants were virtually screened using molecular docking, pharmacokinetic and toxicological analysis. Ten phytoconstituents (L82, L86, L92, L121, L148, L187, L211, L221, L228) exhibited their high binding affinity with BuChE, and five phytoconstituents, namely L119, L147, L149, L192 and L193, exhibited their strong binding ability with AChE. Subsequently, these phytoconstituents were evaluated for their ADMET properties. As result, L221 is predicted to be highly bioavailable and readily absorbed by the human intestinal tract without significant toxicity concerns, making it suitable for oral administration. Crucially, it can penetrate the blood-brain barrier (BBB), allowing it to effectively reach the central nervous system. Molecular dynamics simulations and MM-PBSA analysis revealed that the best-screened phytoconstituent form thermodynamically favorable and stable complex with the BuChE binding site. The conducted investigations highlighted promising outcomes that can orient towards the rational development of effective Cholinesterase inhibitors.

## 1 Introduction

Medicinal plants have been used for centuries, in traditional medicine, to treat various diseases, including tumors, heart diseases and diabetes [[1], [2], [3], [4]]. They have also been used to treat common ailments such as cold, coughs, and headaches [[5], [6], [7]]. Some of the most used medicinal plants include aloe vera, chamomile, echinacea, ginkgo biloba and ginseng. Each of these plants has unique properties that make them effective in treating specific conditions. Aloe vera, for example, is known for its ability to soothe and heal skin irritations and burns [8,9]. Chamomile is widely used for its therapeutic properties to treat digestive issues, anxiety, cramps and dermatitis [10,11], while echinacea is used to boost the immune system [12]. Ginkgo biloba can enhance memory and cognitive function [13,14], while ginseng is employed to increase energy and reduce stress [15].

Medicinal plants can be found all over the world and have been used by different cultures for different purposes. Morocco is known for its rich flora englobing a diverse range of medicinal plants. Moroccan medicinal plants are a valuable resource for traditional healers and modern medicine alike [16,17]. Recent studies confirm the bioactive potential of Moroccan plants. Tazi et al., demonstrated the antioxidant and antimicrobial activities of *Cymbopogon citratus* L cultivated under North African conditions in Morocco [18]. *Retama dasycarpa* is another Moroccan plant exhibiting promising antioxidant and antimicrobial activities [19]. Other studies highlighted the anti-inflammatory and analgesic properties of Moroccan medicinal plants [20].

Medicinal plants contain a wide range of phytoconstituents, known as secondary metabolites, that are responsible for their therapeutic properties. Secondary metabolites are organic compounds classified into various families. They include alkaloids, flavonoids, terpenoids, and phenolic compounds. Alkaloids are nitrogen-containing compounds, such as caffeine, nicotine, and morphine. They can act as anti-tumor and anti-inflammatory agents [21,22].

Terpenoids are organic compounds derived from isoprene units and are commonly found in many plants. They have various biological activities, including anti-inflammatory, anti-tumor, and anti-microbial properties [23,24]. Phenolic compounds, which are potent antioxidant agents, are aromatic compounds derived from phenol. They are found in many fruits and vegetables [25,26].

Alzheimer's disease (AD) is an irreversible neurological disorder affecting the brain function. It is the most common cause of dementia, which is characterized by a progressive impairment of communication skills, memory and social activities [27]. AD is named after the German physician Alois Alzheimer who identified the disorder in 1906 [28]. Acetylcholine is an important neurotransmitter for memory and learning. During the AD progression, the levels of acetylcholine decrease due to the loss of cholinergic neurons. Cholinesterase inhibitors (AChEIs) are a class of drugs used to ameliorate the cognitive function in patients with AD. These drugs inhibit the enzymatic activity of acetylcholinesterase (AChE) and butyrylcholinesterase (BuChE), which breaks down the neurotransmitter acetylcholine (ACh) in the brain [29,30].

Moroccan flora is renowned for its remarkable biodiversity and the abundance of various medicinal plants that have been utilized in traditional medicine for centuries. Despite their significant potential, many of these plants have not undergone comprehensive scientific investigation to explore their pharmacological properties, positioning them as promising candidates for novel drug discovery initiatives. Therefore, this study seeks to delve into the potential of selected Moroccan medicinal plants as inhibitors of cholinesterase enzymes.

To achieve this objective, a range of secondary metabolites extracted from these medicinal plants have been examined using advanced computer-aided drug design techniques [[31], [32], [33]]. This research not only aims to uncover new therapeutic agents but also contributes to the valorization of specific Moroccan plants, highlighting their relevance as potential drug candidates for the symptomatic treatment of AD.

However, it is crucial to note that the findings obtained from in silico studies require further validation through rigorous in vitro and in vivo experiments. Such validation is essential to confirm the biological activity of the identified compounds and to assess their efficacy and safety for potential therapeutic applications.

## 2 Materials and methods

### 2.1 General approach

Virtual screening is a computational process aiming to explore potential drug candidates. The first step in virtual screening is the determination of the tridimensional structure of a target protein using characterization techniques such as X-ray crystallography or NMR spectroscopy. Once the structure is identified, modeling techniques such as molecular docking and molecular dynamics simulations are used to highlight relevant medication candidates having the ability to interact with the biological target and modulate its activity. The most promising candidates are then further evaluated for their drug likeness and ADMET proprieties.

### 2.2 Biological target

Cholinesterase enzymes (AChE and BChE) regulate the acetylcholine levels in the brain, which is involved in many physiological processes, including muscle contraction, heart rate, and cognitive function [[34], [35], [36]].

Cholinesterase inhibitors are commonly used to treat AD. These drugs work by improving the transmission of signals between nerve cells in the brain, which can help to slow down the progression of AD. By inhibiting the breakdown of acetylcholine, these drugs leads to improved cognitive function and muscle control [31,37,38].

The current study attempts to identify potential cholinesterase inhibitors from natural products. The tridimensional structures of the target enzymes were retrieved from the Protein Data Bank available online at http://www.rcsb.org (BuChE PDB ID: 6eul; AChE PDB ID: 4ey7).

### 2.3 Molecular docking analysis

Molecular docking has become a crucial method for drug discovery, as it can help highlight promising drug candidates having the ability to bind to a specific target protein with high affinity and specificity [[39], [40], [41], [42], [43], [44], [45]]. In the current work, AutoDock vina software [46] was exploited to predict the binding mode and affinity of natural products towards the cholinesterase enzymes. The biological target structures were initially prepared by eliminating all water molecules and crystalized ligands, adding missing atoms, and further adding Kollman charges using AutoDockTools [47]. Besides, docking boxes were defined around the protein binding sites, to delimit the region within the receptor where the ligands will be docked. The coordinates of the grid boxes corresponding to BuChE and AChE are (x = 42.834, y = 19.853, z = 24.398) and (X = −14.108, Y = −43.833, Z = 27.670), respectively. The axe sizes of the docking boxes were set to: OX = 40 Å, OY = 40 Å and OZ = 40 Å. The protein structures have been regarded as rigid, while the ligand structures have been deemed flexible. Other parameters, including exhaustiveness (Exh), energy range (ER), and number of binding modes (NBM), have been assigned default values (Exh = 8; ER = 4; NBM = 9). Following that and before starting the docking study, the ligand structures were also prepared as described below. Finally, the interactions maintaining the docked ligands inside the binding site corresponding to the biological targets were highlighted through Discovery Studio 2021 software [48].

### 2.4 Ligand preparation

Various secondary metabolites extracted from twenty-eight medicinal plants belonging to eight plant families including Lamicaceae, Rosaceae, Zingiberaceae, Apiaceae, Asteraceae, Lauraceae, Fabaceae and Myrtaceae, were collected from previous phytochemical studies [[49], [50], [51], [52], [53], [54], [55], [56], [57], [58], [59], [60], [61], [62], [63], [64], [65], [66], [67], [68], [69], [70], [71], [72], [73]]. These compounds were carefully selected for their chemical diversity and their origin from plant species that have not previously been investigated for their potential therapeutic effects on Alzheimer's disease.

Table S1 (Supplementary material) includes the structures and the systematic or trivial names corresponding to the collected phytoconstituents. To ensure that they are in their most stable conformations, the phytoconstituents structures were optimized using the MMFF94 force field with the steepest Descent algorithm implemented in Avogadro software [74]. After that, the ligand structures were prepared for docking by adding Gasteiger charges, and merging polar hydrogens using AutoDockTools.

### 2.5 ADMET and drug-likeness properties

ADMET analysis predict how a drug is absorbed, distributed, metabolized, eliminated by the body, and whether it has any potential toxicity [75]. Drug-likeness properties refer to the characteristics that make a molecule more likely to be a successful drug candidate. Understanding pharmacokinetic, pharmacodynamic and drug-likeness properties is essential for drug discovery and development, as it helps researchers identify molecules that have the potential to become successful drugs. In the current study, ADMET and drug-likeness properties were evaluated using pKCSM server [76].

### 2.6 Molecular dynamics simulations

In order to simulate the molecular dynamics of the studied ligand-protein complexes, we used GROMACS [77] software with charmm27force field [78]. The ligands topologies were produced using SwissParam server [79]. A dodecahedral simulation box (box volume: 675.01 nm^3^) was created to encompass the studied systems and subsequently TIP3P water molecules were added to form an aqueous environment inside the simulation box. The electrical neutrality of the studied systems was ensured by adding Sodium and chloride ions. Thereafter, the systems optimization was done by applying the steepest descent method. Following that, to prepare the simulation conditions, the investigated systems were undergone NVT equilibration at 300 K for 1 ns followed by NPT equilibration utilizing Parrinello-Rahman barostat at 1 atm for 1 ns [80]. After the equilibration stage, the molecular dynamics of the studied systems were simulated in aqueous medium during 150 ns with a time step of 2 fs. Finally, the trajectories of various parameters including RMSD, RMSF, RoG, number of hydrogen bonds, and SASA, were extracted from the MD simulation.

### 2.7 Binding energy calculation

Molecular mechanics Poisson–Boltzmann surface area (MM-PBSA) is a popular computational approach used in molecular modeling to estimate the interaction free energies. In the current investigation, after performing MD simulations, g_mmpbsa tool [77], which implements the MM-PBSA method, was used to calculate the binding free energy corresponding to the studied ligands.

## 3 Results and discussion

### 3.1 Re-docking

Re-docking is a computational validation technique used to assess the accuracy of docking methods by reintroducing a known ligand into its target protein's binding site and comparing the predicted binding pose with the experimentally determined one. The predicted pose is compared to the experimentally obtained pose using metrics like Root Mean Square Deviation (RMSD).

**Fig. 1:**
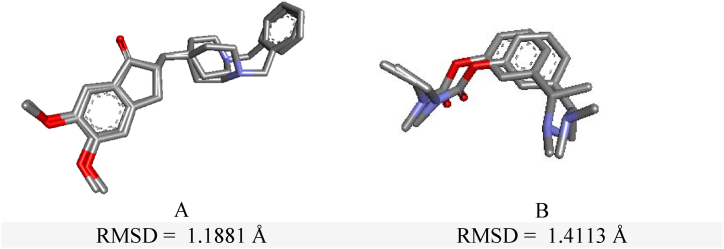
A- Redocking of Donepezil. B- Redocking of Rivastigmine.

In the current study, AChE is co-crystallized with Donepezil and BuChE is crystallized with Rivastigmine. The re-docked conformations of the co-crystallized ligands were superimposed on their original conformations and the RMSD values was calculated (Fig. 1(A and B)). The calculated RMSD values are inferior to 2 Å, confirming the reliability of the docking protocol for further use in drug discovery or molecular interaction studies.

### 3.2 Molecular docking results

Rivastigmine, Galantamine and Donepezil, which are cholinesterase inhibitors approved by the Food and Drug Administration, were also docked to serve as references. The binding energies values corresponding to the investigated phytoconstituents are presented in Table 1.

**Table 1:** Binding energies (BE) relative to the docked ligands (lig).

BE (Kcal/mol)			BE (Kcal/mol)			BE (Kcal/mol)			BE (Kcal/mol)			BE (Kcal/mol)		
Lig	BuChE	AChE	Lig	BuChE	AChE	Lig	BuChE	AChE	Lig	BuChE	AChE	Lig	BuChE	AChE
L1	−4.6	−5.1	L53	−6.6	−6.6	L104	−6.9	−8.2	L155	−6.4	−7.2	L206	−6.6	−7.3
L2	−4.9	−5.5	L54	−6.4	−7.3	L105	−8.9	−9.8	L156	−6.5	−7.1	L207	−9.9	−10.2
L3	−6.4	−6.6	L55	−10.4	−9.3	L106	−7.4	−7.7	L157	−10.2	−10.0	L208	−9.5	−9.2
L4	−7.1	−8.7	L56	−6.8	−7.4	L107	−8.5	−9.0	L158	−9.7	−8.1	L209	−9.6	−9.3
L5	−6.5	−6.8	L57	−6.5	−7.5	L108	−10.4	−8.7	L159	−6.6	−7.2	L210	−9.2	−9.3
L7	−5.1	−6.6	L58	−8.1	−8.2	L109	−8.9	−8.7	L160	−6.3	−7.3	L211	−11.7	−9.3
L8	−4.8	−5.4	L59	−8.6	−9.0	L110	−6.4	−6.6	L161	−6.5	−6.7	L212	−6.5	−6.8
L9	−5.4	−6.1	L60	−9.1	−9.5	L111	−6.3	−7.0	L162	−6.5	−6.9	L213	−8.3	−9.1
L10	−7.0	−7.2	L61	−8.6	−8.7	L112	−5.9	−7.0	L163	−8.1	−9.5	L214	−9.1	−10.2
L11	−6.2	−6.9	L62	−8.3	−10.0	L113	−5.9	−7.7	L164	−8.1	−8.6	L215	−7.0	−7.6
L12	−7.5	−8.4	L63	−6.5	−7.1	L114	−6.9	−7.7	L165	−5.7	−6.7	L216	−6.0	−6.7
L13	−5.2	−5.7	L64	−8.6	−9.0	L115	−8.5	−9.9	L166	−9.9	−10.1	L217	−6.1	−6.8
L14	−5.3	−5.8	L65	−6.5	−7.1	L116	−8.4	−8.7	L167	−5.9	−6.6	L218	−9.7	−7.7
L15	−5.2	−5.7	L66	−6.1	−6.9	L117	−6.5	−6.9	L168	−6.2	−6.7	L219	−6.2	−7.4
L16	−7.7	−8.5	L67	−5.9	−6.6	L118	−9.9	−8.4	L169	−7.0	−7.9	L220	−8.5	−9.0
L17	−6.0	−6.6	L68	−5.8	−6.5	L119	−10.6	−11.6	L170	−5.9	−7.3	L221	−11.5	−8.8
L18	−5.6	−6.2	L69	−6.3	−7.3	L120	−4.5	−4.7	L171	−6.1	−6.6	L222	−4.7	−5.5
L19	−5.9	−6.3	L70	−6.5	−7.8	L121	−11.9	−9.9	L172	−6.7	−8.0	L223	−8.1	−9.8
L20	−5.7	−6.4	L71	−6.1	−7.1	L122	−8.6	−8.4	L173	−6.5	−7.7	L224	−6.3	−7.4
L21	−9.9	−10.3	L72	−6.9	−7.4	L123	−8.6	−8.8	L174	−6.1	−7.1	L225	−5.7	−7.5
L22	−9.4	−9.8	L73	−7.1	−7.4	L124	−4.6	−5.0	L175	−6.2	−7.4	L226	−11.0	−10.2
L23	−9.3	−8.8	L74	−9.4	−9.4	L125	−10.6	−10.2	L176	−6.4	−7.2	L227	−6.2	−6.7
L24	−7.0	−8.1	L75	−6.5	−7.4	L126	−5.4	−6.6	L177	−6.0	−7.5	L228	−11.8	−10.2
L25	−6.2	−7.2	L76	−8.3	−9.3	L127	−6.4	−8.1	L178	−5.0	−5.6	L229	−6.5	−7.4
L26	−9.3	−9.2	L77	−8.2	−8.7	L128	−5.9	−6.9	L179	−5.5	−6.1	L230	−6.5	−7.0
L27	−5.3	−5.9	L78	−4.8	−5.6	L129	−8.9	−8.7	L180	−5.9	−7.1	L231	−6.6	−7.2
L28	−6.3	−7.1	L79	−8.5	−8.8	L130	−8.7	−9.3	L181	−6.1	−6.9	L232	−6.7	−7.0
L29	−9.6	−10.3	L80	−4.3	−4.9	L131	−8.3	−9.5	L182	−5.9	−7.1	L233	−6.5	−7.4
L30	−9.8	−10.1	L81	−6.6	−7.4	L132	−7.1	−7.5	L183	−8.5	−8.5	L234	−7.3	−8.7
L31	−6.2	−7.4	L82	−10.3	−10.4	L133	−7.1	−7.5	L184	−5.8	−6.7	L235	−6.4	−7.0
L32	−8.3	−8.6	L83	−5.1	−6.0	L134	−5.4	−6.2	L185	−4.6	−5.3	L236	−8.8	−9.2
L33	−7.8	−8.7	L84	−5.6	−6.3	L135	−8.1	−8.3	L186	−4.8	−5.6	L237	−6.5	−8.2
L34	−10.0	−8.5	L85	−5.2	−6.0	L136	−6.5	−7.4	L187	−11.5	−9.1	L238	−5.1	−5.9
L35	−6.2	−7.4	L86	−11.4	−10.5	L137	−8.4	−8.3	L188	−6.3	−7.5	L239	−5.6	−6.7
L36	−7.9	−9.1	L87	−9.1	−9.8	L138	−9.5	−9.5	L189	−6.1	−7.0	L240	−7.3	−7.7
L37	−7.6	−8.1	L88	−5.7	−7.1	L139	−9.4	−9.6	L190	−5.8	−6.1	L241	−5.8	−6.6
L38	−7.8	−8.6	L89	−6.0	−6.5	L140	−9.4	−9.6	L191	−6.6	−7.4	L242	−8.6	−8.9
L39	−10.1	−9.7	L90	−6.8	−8.0	L141	−6.3	−7.5	L192	−10.0	−11.0	L243	−6.3	−6.8
L40	−8.4	−8.8	L91	−7.4	−8.1	L142	−6.2	−7.2	L193	−9.5	−10.5	L244	−6.1	−6.3
L41	−8.1	−8.5	L92	−10.1	−10.4	L143	−6.1	−6.5	L194	−6.3	−7.3	L245	−8.6	−9.3
L42	−6.2	−6.5	L93	−5.9	−7.5	L144	−6.5	−7.2	L195	−7.1	−7.6	L246	−9.9	−9.8
L43	−7.2	−7.7	L94	−6.1	−7.0	L145	−6.6	−7.4	L196	−7.0	−7.5	L247	−9.7	−9.1
L44	−6.6	−6.8	L95	−6.2	−7.0	L146	−6.5	−7.9	L197	−8.4	−9.3	L248	−8.2	−8.0
L45	−8.0	−8.4	L96	−5.6	−6.9	L147	−10.5	−11.3	L198	−9.6	−10.2	L249	−7.8	−8.7
L46	−5.2	−6.0	L97	−6.3	−6.8	L148	−11.1	−9.6	L199	−6.6	−8.6	L297	−8.3	−9.1
L47	−8.2	−8.7	L98	−8.6	−9.4	L149	−9.8	−10.4	L200	−6.9	−7.5	L298	−8.5	−8.9
L48	−6.7	−6.9	L99	−7.5	−7.8	L150	−6.1	−7.1	L201	−6.5	−7.4	L299	−8.4	−8.5
L49	−8.2	−8.6	L100	−6.5	−7.3	L151	−8.9	−10.0	L202	−6.6	−6.8			
L50	−8.4	−8.7	L101	−6.8	−8.7	L152	−9.3	−10.1	L203	−9.4	−8.7			
L51	−10.4	−9.1	L102	−6.3	−7.2	L153	−6.3	−6.7	L204	−6.4	−6.6			
L52	−6.2	−6.6	L103	−6.6	−7.2	L154	−6.6	−7.2	L205	−7.4	−8.0			
Gal	−9.3	−9.7	Gal	−9.3	−9.7	Gal	−9.3	−9.7	Gal	−9.3	−9.7	Gal	−9.3	−9.7
Riv	−7.3	−8.1	Riv	−7.3	−8.1	Riv	−7.3	−8.1	Riv	−7.3	−8.1	Riv	−7.3	−8.1
Don	−10.0	−11.4	Don	−10.0	−11.4	Don	−10.0	−11.4	Don	−10.0	−11.4	Don	−10.0	−11.4

The docking outcomes revealed that all docked ligands exhibited negative binding energies, revealing that the recognition process between the target proteins (AChE and BuChE) and the investigated phytoconstituents is thermodynamically favorable. Taking into consideration the binding energies showed by Rivastigmine (Riv), Galantamine (Gal) and Donepezil (Don), fifteen phytoconstituents were found to be able to strongly binds to the binding sites of the target proteins, limiting their biological activities. Among them, ten phytoconstituents (L82, L86, L92, L121, L148, L187, L211, L221, L226, L228) exhibited their high binding affinity with BuChE. Indeed, their predicted binding energies are lower than those exhibited by the reference drugs (BE(Gal) = −9.3 kcal/mol, BE(Riv) = −7.3 kcal/mol, BE(Don) = −10.00 kcal/mol). Five phytoconstituents, namely L119, L147, L149, L192 and L193, exhibited their strong binding ability with AChE. They have been predicted to possess lower binding energy than those exhibited by Gal and Riv (BE(Gal) = −9.7 kcal/mol, BE(Riv) = −8.1 kcal/mol). Indeed, the more negative the binding energy, the stronger the ligand-protein binding. Table 2 present the structures corresponding to the phytoconstituents having exhibited high binding affinity.

**Table 2:** Structures of phytoconstituents having exhibited the highest binding affinity.

Lig	Structure	Lig	Structure	Lig	Structure
L82		L86		L92	
L119		L121		L147	
L148		L149		L187	
L192		L193		L211	
L221		L226		L228	

### 3.3 Drug-likeness properties

As described by Lipinski and Veber [81,82], bioavailable compounds must have the following properties:

⁃ Molecular weight (MW) is equal or smaller than 500 Da (MW);
⁃ Hydrogen bond acceptors (HBA) are equal or smaller than ten atoms;
⁃ Hydrogen bond donors (HBD) are equal or smaller than five atoms;
⁃ Partition coefficient (Log P) is equal or smaller than five;
⁃ Polar area (PSA) is equal or smaller than 140 Å^2^;
⁃ Rotatable bonds (RB) is equal or smaller than ten.

In the present study, the rules of Lipinski and Veber were adopted to evaluate the drug likeness properties characterizing the best docked ligands (L82, L86, L92, L119, L121, L147, L148, L149, L187, L192, L193, L211, L221, L226, L228). Table 3 summarizes the predicted properties. According to the obtained outcomes, L86, L92, L211 and L228 are predicted to be non-bioavailable drug candidate contrary to the remaining ligands, which limits their future use as orally administered drugs.

**Table 3:** Molecular descriptors of studied phytoconstituents.

Ligand	Lipinski and Veber rules						Number of violations
	MW (Da)	Log P	RB	HBA	HBD	PSA (Å^2^)	
L 82	306.232	−0.064	3	4	2	144.830	1
L 86	543.328	0.276	7	14	7	227.393	4
L 92	435.276	0.596	4	11	7	180.823	3
L 119	306.232	1.400	1	4	2	140.522	0
L 121	426.342	0.832	1	3	2	205.515	1
L 147	401.262	0.785	4	9	5	171.235	1
L 148	377.337	1.929	1	1	1	192.398	1
L 149	280.191	1.083	1	6	4	117.313	1
L 187	136.109	0.253	4	1	0	70.185	0
L 192	321.223	−0.263	3	5	1	147.278	1
L 193	274.234	0.517	4	2	2	136.560	0
L 211	435.276	−0.730	0	10	6	178.533	2
L 221	192.129	1.158	4	3	0	90.272	0
L 226	365.326	2.092	5	1	1	186.349	1
L 228	577.442	1.401	4	6	1	274.567	2
Donepezil	350.269	1.635	6	4	0	167.005	1
Galantamine	255.188	0.822	1	4	1	118.844	0
Rivastigmine	228.166	0.722	4	3	0	109.146	0

Threshold							

MW ≤ 500		LogP ≤ 5	RB ≤ 10	HBA ≤ 10	HBD ≤ 5	PSA ≤ 140	N.Viol ≤ 1

### 3.4 ADMET properties

According to Table 4, six phytoconstituents (L119, L148, L187, L221, L226 and L228) are expected to be highly absorbable by the human intestine (HIA ≥ 90 %). Furthermore, except the remaining compounds, L119, L148, L187, L193, L221 and L226 are predicated to be permeable to the Caco-2 cell line, confirming their high intestinal permeability [76].

**Table 4:** Absorption and distribution indices calculated for the studied phytoconstituents.

Ligand	Absorption				Distribution	
	Caco2	HIA (%)	P-gp substrate	P-gp Inhibitor I/II	BBB permeability (log BB)	CNS permeability (log PS)
L 82	0.635	68.171	Yes	No/No	0.063	−2.953
L 86	0.502	60.321	Yes	No/No	−1.880	−4.327
L 92	−0.917	45.437	Yes	No/No	−1.761	−4.206
L 119	1.165	98.884	Yes	No/No	−0.006	−2.365
L 121	−0.112	50.336	No	No/No	0.260	−2.411
L 147	0.290	42.605	Yes	No/No	−1.126	−3.651
L 148	1.249	100	No	No/Yes	1.239	−1.704
L 149	−0.400	69.492	Yes	No/No	−1.140	−2.662
L 187	1.119	100	No	No/No	0.266	−2.358
L 192	0.649	76.669	No	No/No	−0.150	−2.980
L 193	1.227	64.626	Yes	No/No	0.237	−2.107
L 211	0.496	63.585	Yes	No/No	−1.264	−3.677
L 221	1.722	91.104	No	No/No	0.186	−2.015
L 226	1.237	100	No	No/Yes	1.152	−2.018
L 228	0.259	100	No	Yes/Yes	−0.112	−2.990
Donepezil	1.262	99.816	Yes	Yes/Yes	0.107	−1.422
Galantamine	1.749	92.857	No	No/No	−0.058	−2.808
Rivastigmine	1.741	98.088	No	No/No	−0.005	−2.751

P-glycoprotein (P-gp) is an ATP-binding cassette (ABC) transporter functioning as a biological barrier by expelling toxins and xenobiotics out of cells. As depicted in Table 4, the compounds L121, L148, L187, L192, L221, L226 and L228 are not P-gp substrates, meaning that they cannot be expelled out of cells by the P-gp. On the other hand, L148, L228 and L228 are predicted to be P-gp inhibitors, indicating that they can perturb the normal functioning of P-gp.

The blood-brain barrier (BBB) is a biological barrier protecting the brain from exogenous compounds. The drug candidates for the AD treatment should be BBB permeable. According to the obtained results, L119, L148, L187, L193, L221 and L226 are predicted to be permeable to the BBB and can penetrate the Central Nervous System (CNS) [76].

Cytochrome P450's (CYP) are enzymes implicated in the metabolism of several medications. Therefore, CYP inhibitors can affect the metabolism of these drugs. According to Table 5, L119, L121, L148, L192, L226 and L228 are likely to be metabolized by CYP3A4. Besides, no tested compound is able to be CYP inhibitors, indicating that they are unable to affect the metabolism of other drugs.

**Table 5:** Metabolism and excretion indices calculated for the studied phytoconstituents.

Ligand	Metabolism				Excretion	
	CYP2D6 substrate	CYP3A4 substrate	CYP2D6 inhibitor	CYP3A4 inhibitor	Total Clearance	Renal OCT2 substrate
L 82	No	No	No	No	1.240	No
L 86	No	No	No	No	0.827	No
L 92	No	No	No	No	0.593	No
L 119	No	Yes	No	No	0.888	No
L 121	No	Yes	No	No	1.245	No
L 147	No	No	No	No	0.760	No
L 148	No	Yes	No	No	1.385	No
L 149	No	No	No	No	0.668	No
L 187	No	No	No	No	1.278	No
L 192	No	Yes	No	No	0.953	No
L 193	No	No	No	No	1.371	No
L 211	No	No	No	No	0.418	No
L 221	No	No	No	No	0.903	No
L 226	No	Yes	No	No	1.588	No
L 228	No	Yes	No	No	1.487	No
Donepezil	Yes	Yes	Yes	Yes	1.430	Yes
Galantamine	No	No	No	No	1.203	No
Rivastigmine	No	No	No	No	0.569	No

Organic Cation Transporter 2 (OCT2) is a biological transporter involved in renal clearance of medications and endogenous compounds. According to the obtained results, no investigated phytoconstituent is likely to be OCT2 substrate.

Besides, no investigated phytoconstituent is likely to be mutagenic (Table 6). In addition, except L148 and L193, all the investigated phytoconstituents are not hepatotoxic and they are unable to produce Skin Sensitization effects. Furthermore, L148 and L226 are predicted to be hERG II inhibitors, indicating that they can lead to a life-threatening ventricular arrhythmia by affecting the electrical activity of the heart.

**Table 6:** Toxicity indices calculated for the studied phytoconstituents.

Ligand	Toxicity							
	AMES toxicity	Max. tolerated dose (human)	hERG I inhibitor	hERG II inhibitor	LD50	LOAEL	Hepatotoxicity	Skin Sensitization
L 82	No	−0.715	No	No	1.795	2.563	No	No
L 86	No	0.252	No	No	2.689	3.623	No	No
L 92	No	0.319	No	No	2.771	4.891	No	No
L 119	No	−0.362	No	No	2.023	1.431	No	No
L 121	No	0.375	No	No	2.133	2.415	No	No
L 147	No	0.040	No	No	2.345	4.379	No	No
L 148	No	−0.852	No	Yes	2.203	0.064	Yes	No
L 149	No	0.552	No	No	2.437	2.379	No	No
L 187	No	0.790	No	No	1.316	0.804	No	No
L 192	Yes	−0.377	No	No	2.129	1.626	No	No
L 193	No	−0.936	No	No	1.474	2.578	No	Yes
L 211	No	0.319	No	No	2.591	3.236	No	No
L 221	No	0.877	No	No	1.557	2.260	No	Yes
L 226	No	−0.937	No	Yes	1.646	0.047	No	No
L 228	No	0.157	No	No	2.320	0.714	No	No
Donepezil	No	−0.413	No	Yes	2.484	2.054	No	No
Galantamine	No	−0.303	No	No	2.546	1.011	No	No
Rivastigmine	No	0.238	No	No	2.397	0.764	No	No

Considering its ADMET and drug likeness profiles, L221 is highly recommended as potent drug candidate. It is predicted to be highly bioavailable and readily absorbed by the human intestinal tract, making it suitable for oral administration. Crucially, L221 can penetrate the blood-brain barrier (BBB), allowing it to effectively reach the central nervous system, which is vital for addressing neurodegenerative diseases like Alzheimer's. Its favorable profile indicates that it has the potential to deliver therapeutic effects without significant toxicity concerns. Therefore, L221 could potentially lead to significant therapeutic benefits for Alzheimer's patients, positioning it as a top choice for further development and research.

### 3.5 Ligand-protein interactions

Rivastigmine is a medication that is commonly administrated to treat AD symptoms. It works by inhibiting the BuChE enzyme, which is responsible for breaking down the neurotransmitter acetylcholine in the brain.

When Rivastigmine is administered, it binds to the BuChE enzyme. This binding prevents the enzyme from functioning properly, inhibiting its ability to break down acetylcholine. As a result, the levels of acetylcholine in the brain increase. By inhibiting the enzymatic activity of BuChE, Rivastigmine helps to enhance the cholinergic transmission in the brain, which could enhance memory and cognitive function in Alzheimer's patients.

**Fig. 2:**
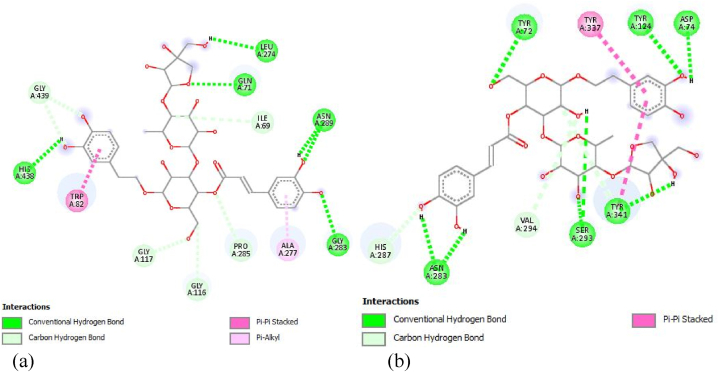
(a) - 2D Diagram depicting the L221-BuChE interactions. (b) - 2D Diagram depicting the L221-AChE interactions.

**Fig. 3:**
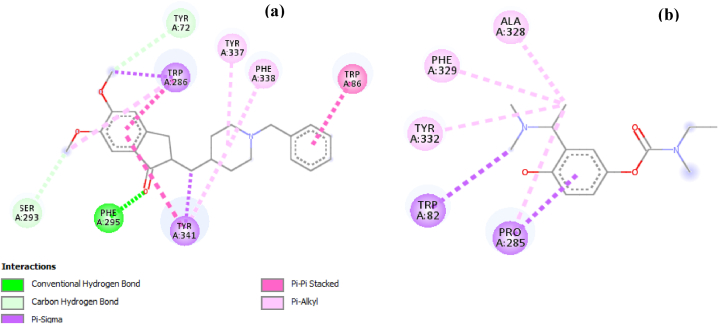
The Donepezil-AChE interactions (A) and Rivastigmine-BuChE interactions (B) in bidimensional diagram.

Adopting analogical reasoning, a ligand that can overlap the active site of the BuChE enzyme by forming a sable complex, can become a potential BuChE inhibitor. In the current study, L221 was predicted to present high binding affinity towards the BuChE and highlighted to possess a good ADMET profile. Besides, the molecular interactions keeping L221 docked inside the BuChE binding site were further highlighted to better understand the occurred molecular recognition process. Fig. 2-A depict the involved interactions. The studied ligand (L221) has been complexed in the BuChE binding site by involving various non-covalent interactions, including hydrophobic contacts as well as hydrogen bonds. Indeed, as summarized in Table 7, L221 is docked at the BuChE pocket through fourteen hydrogen bonds mediated by ten residues, namely GLN71, GLY 283, ASN 289, LEU 274, HIS 438, GLY 117, PRO 285, GLY 439, ILE69 and GLY 116, in addition to two Pi-Pi Stacked interactions with TRP 82 and a Pi-Alkyl interaction with ALA 277. Moreover, the implicated interactions are well distributed over the ligand's backbone, stabilizing the L221 structure inside the binding site of BuChE. In addition, the involved interactions appeared over very short distances, which reinforces the coherence of the formed complex. Besides, Rivastigmine binds to BuChE by involving hydrophobic interactions with residues like ALA A328, PHE A329, TYR A332, TRP A82, and PRO A285 (Fig. 3-A), which could be vital in influencing BuChE's enzymatic activity. Taking into consideration the above-mentioned description, the molecular recognition between L221 and BuChE can lead to stable complex. To confirm the complex stability, it is necessary to simulate its molecular dynamics over a period of time.

**Table 7:** Receptor-ligand interactions in three-dimensional space.

Ligand	BuChE residues	Category	Distance (Å)
L221	GLN 71	Hydrogen bond	2.482
	GLY 283	Hydrogen bond	2.985
	ASN 289	Hydrogen bond	2.224
		Hydrogen bond	2.953
		Hydrogen bond	1.993
	LEU 274	Hydrogen bond	2.603
		Hydrogen bond	2.535
	HIS 438	Hydrogen bond	2.574
	GLY 117	Hydrogen bond	3.588
	PRO 285	Hydrogen bond	3.307
	GLY 439	Hydrogen bond	3.372
		Hydrogen bond	3.429
	ILE 69	Hydrogen bond	3.720
	GLY 116	Hydrogen bond	3.166
	TRP 82	Hydrophobic	3.859
		Hydrophobic	3.896
	ALA 277	Hydrophobic	4.585

On the other hand, L221 was complexed into the AChE binding site by involving various non-covalent interactions, including hydrophobic contacts as well as hydrogen bonds (Fig. 2-B). Indeed, it is docked to the AChE pocket by involving ten hydrogen bonds with TYR 72, TYR 124, ASP 74, TYR 341, SER 293, VAL 294, ASN 283 and HIS 287, in addition to two Pi-Pi Stacked interactions with TYR 337 and TYR 341. In contrast, as described in our previous studies [40,41,71,80,81], Donepezil binds to AChE by interacting with TRP A286, TYR A72, TYR A337, PHE A338, TRP A86, TYR A341, PHE A295, and SER A293 (Fig. 3-B). These specific residues may be fundamental in influencing AChE's enzymatic functions.

### 3.6 Molecular dynamics simulations

**Fig. 4:**
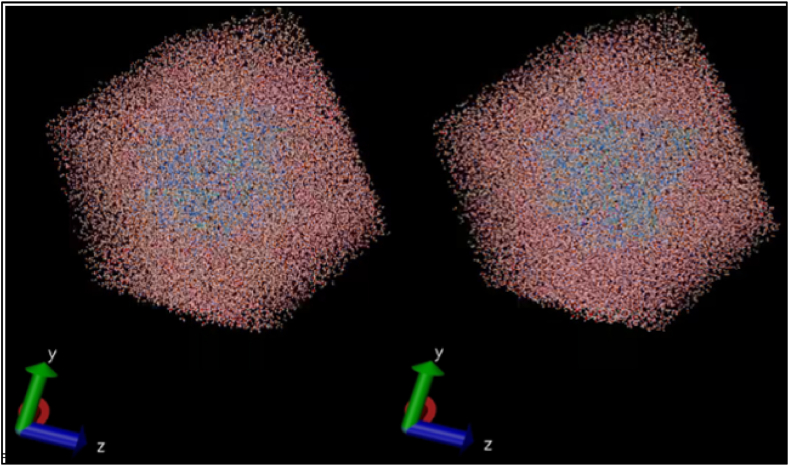
Images illustrating the performed simulation.

**Fig. 5:**
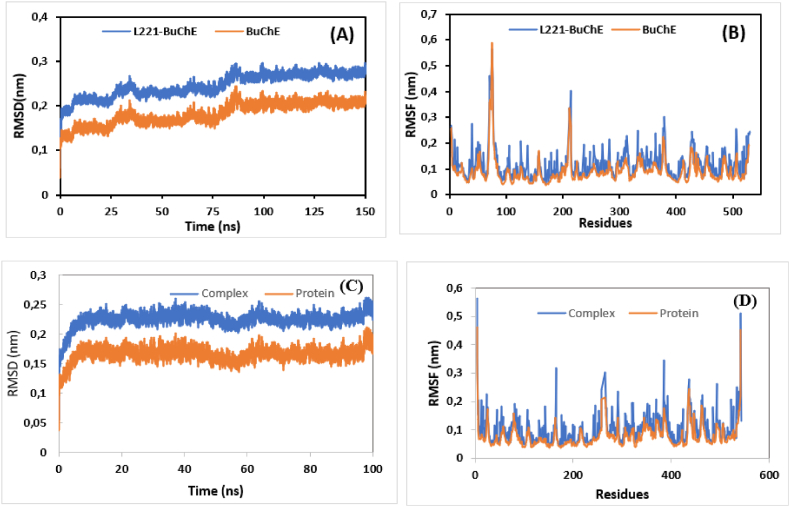
A- RMSD of the backbone of BuChE and L221-BuChE complex as a function of time. B- RMSF of Cα atoms of BuChE in the absence and presence of L221. C- RMSD of the backbone of AChE and L221-AChE complex as a function of time. D- RMSF of Cα atoms of AChE in the absence and presence of L221.

The molecular dynamics of BuChE alone and L221-BuChE complex have been simulated over 150 ns to gain insight into their dynamical behavior in an aqueous medium. Fig. 4 shows some images illustrating the performed simulation. Initially, the stability of the studied systems was investigated by computing their root mean square deviation (RMSD) as expressed in Fig. 5-A. The RMSD of all systems fluctuated until reaching a relatively steady state, with negligible variation at some intervals, indicating that the studied systems adapt to the simulation conditions before reaching a steady state. The average RMSD values of BuChE and L221-BuChE complex were predicted to be 0.182 and 0.245 nm, respectively. In addition, it can be noted that the RMSD values corresponding to BuChE alone are lower than those of its relative complex due to the molecular interactions implicated between L221 and its receptor. It was noted also that the RMSD of the studied complex oscillates weakly around 0.23 nm, reflecting the stability of the ligand-BuchE interactions.

The root means square fluctuation (RMSF) has also been computed to investigate the flexibility of the protein residues. Stable regions are characterized by lower RMSF values while increasing RMSF values lead to higher flexibility. The RMSF graphs of all simulated systems are presented in Fig. 5-B. The average RMSF values of BuChE and L221-BuChE complex were predicted to be 0.092 and 0.121 nm, respectively. The complex exhibited relatively more fluctuations compared to the unbounded BuChE, which is evident considering the dynamic behavior of the ligand. Furthermore, the majority of residues exhibited low RMSF values, leading to stable interactions between the studied ligand and its receptor.

**Fig. 6:**
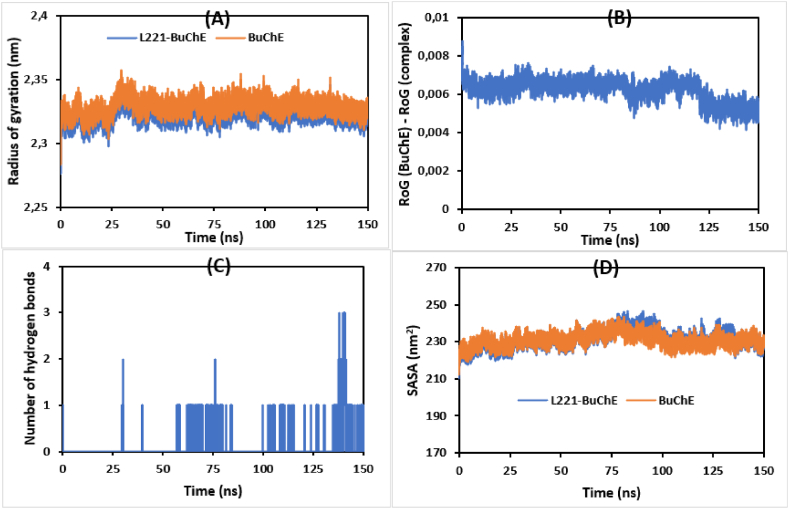
A- RoG of BuChE and L211-BuChE complex as a function of time. B- The Difference between the RoG _(L221-BuChE)_ and RoG _(BuChE)_. C- Number of hydrogens implicated between BuChE and the studied ligand during 150 ns. D- SASA of BuChE and L221-BuChE complex**.**

The radius of gyration (RoG) is another parameter estimated to study the stability of the simulated systems. Fig. 6(A and B) presents the RoG graphs corresponding to the target protein and its complex. The average values of RoG exhibited by BuChE and L221-BuChE complex are predicted to be 2.328 and 2.316 nm, respectively. During the entire MD simulation, the RoG of all systems remained in a steady state, indicating the stability of the simulated complexes in an aqueous medium.

The number of hydrogen bonds formed between the studied ligand (L221) and its target are depicted in Fig. 6-C. The results highlight that some poses of L221 were able to form hydrogen bonds with BuChE,

Solvent accessible surface area (SASA) is another important parameter describing the change in the protein surface area [75]. The SASA trajectories relative to the simulated systems are shown in Fig. 6-D. The average SASA values of BuChE and L221-BuChE complex were predicted to be 230.926 and 231.464 nm^2^, respectively. In addition, we can notice that SASA trajectories remained relatively at a steady state during the entire simulation, indicating the stability of the investigated systems.

**Fig. 7:**
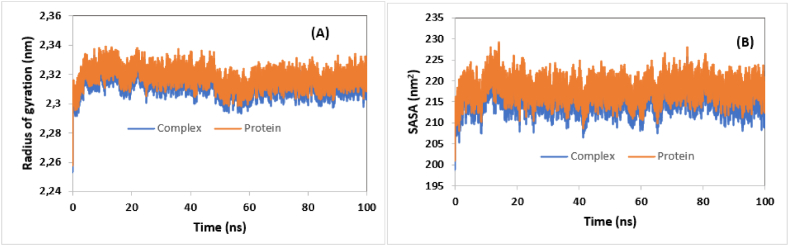
A- RoG of AChE and L211-AChE complex as a function of time. B- SASA of AChE and L221-AChE complex**.**

Overall, the obtained results indicate that the compound L211 forms a stable complex with BuChE, which is significant for inhibiting its biological activity. By binding effectively, L211 may disrupt the BuChE's active site or critical interaction surfaces, leading to reduced catalytic function and impaired interactions with other biomolecules. Indeed, the Root Mean Square Deviation (RMSD) indicates that the complex maintains structural integrity over time, suggesting that L211 binds effectively without inducing large conformational changes in the protein. Furthermore, the root mean square fluctuation (RMSF) reveals no significant changes in flexibility that could affect ligand-protein binding interactions. The Radius of Gyration (RoG) measurements reflect a compact structure of the protein-ligand complex, indicating favorable folding that could enhance stability. Lastly, the Solvent Accessible Surface Area (SASA) analysis shows a reduction in accessible surface area upon L211 binding, leading to a more stable complex. According to Fig. 5(C, D) and Fig. 7(A and B), the same behavior was observed for AChE and L221-AChE complex, reflecting their stability in aqueous environment.

### 3.7 Binding energy results

MM-PBSA is a computational technique widely used in molecular modeling and drug discovery. Its main purpose is to calculate the binding free energy between a ligand and a receptor. In this study, the MM-PBSA method was applied to estimate the binding free energy of L221 and its respective receptor. The results revealed negative binding free energy (Table 8), indicating that the ligand-receptor interactions are thermodynamically favorable and spontaneous. Notably, van der Waals and electrostatic forces contribute to the molecular recognition of BuChE and L221. While polar solvation energy impaired the binding of L221 with BuChE.

**Table 8:** Binding free energy calculated applying the MM-PBSA method.

Energy	Value (kcal/mol)
ΔE_vdW_	−55.64
ΔE_ele_	−50.48
ΔE_PSE_	91.88
ΔE_SASA_	−6.65
ΔG_Total_	−20.88

## 4 Conclusion

In the current study, we attempted to identify potential cholinesterase inhibitors by screening various secondary metabolites via molecular modelling methods. Fifteen phytoconstituents exhibited high affinities towards the target enzymes (BuChE and AChE). However, only one phytoconstituent (L221) was predicted to prosses promising ADMET profile. This phytoconstituent was mainly docked at the BuChE pocket through hydrogen bonds. The MD simulations confirmed that the L221-BuChE interactions in an aqueous medium lead to stable complex inside the binding site of BuChE. The formation of such a complex can perturb the biological function of BuChE and inhibit its ability to break down acetylcholine. The outcome of the current investigation suggests L221 as a promising BuChE inhibitor and it is highly recommended for in-vitro and in-vivo testing.

## CRediT authorship contribution statement

**Hassan Nour:** Writing – review & editing, Writing – original draft, Investigation, Formal analysis, Data curation, Conceptualization. **Oussama Abchir:** Writing – original draft, Data curation, Conceptualization. **Nouh Mounadi:** Conceptualization. **Abdelouahid Samadi:** Visualization, Validation, Funding acquisition. **Belaidi Salah:** Visualization, Validation. **Samir Chtita:** Visualization, Validation, Supervision, Resources, Project administration, Methodology, Investigation.

## Data availability statement

Data included in the article/supplementary material is referenced in the article.

## Declaration of competing interest

The authors declare that they have no known competing financial interests or personal relationships that could have appeared to influence the work reported in this paper.
