# Supplementary material for: Exploration of natural products for the development of promising cholinesterase inhibitors in Alzheimer's disease treatment
Source: Heliyon. 2025 Feb 5;11(4):e42479. doi: 10.1016/j.heliyon.2025.e42479 (PMC11874547; doi:10.1016/j.heliyon.2025.e42479)
Supplement: Multimedia component 1 [file mmc1.docx]

**Exploration of natural products for the development of promising Cholinesterase inhibitors in Alzheimer’s disease treatment**

**Hassan Nour ^(1)^, Oussama Abchir ^(1)^, Nouh Mounadi ^(1)^, Abdelouahid Samadi ^(2, *)^, Belaidi Salah ^(3)^, Samir Chtita ^(1, *)^**

1. Laboratory of Analytical and Molecular Chemistry, Faculty of Sciences Ben M’Sik, Hassan II University of Casablanca, Casablanca, 7955, Morocco
2. Department of Chemistry, College of Science, UAEU, P.O. Box No. 15551, Al Ain, UAE
3. Group of Computational and Medicinal Chemistry, LMCE Laboratory, University of Biskra, BP 145, Biskra 707000, Algeria

^(*)^ Corresponding authors: [samadi@uaeu.ac.ae](mailto:samadi@uaeu.ac.ae); [samirchtita@gmail.com](mailto:samirchtita@gmail.com)

**Supplementary Material**

Table S1: The structures corresponding to phytoconstituents of various medicinal plants.

| **Lamiaceae** | | | | | |
| --- | --- | --- | --- | --- | --- |
| ***Origanum compactum*** | | | | | |
| **42** | **Borneol**  **** | **184** | **Ocimene**    **** | **233** | **Thymol**  **** |
| **56** | **Carvacrol ** | **185** | **Octan-3-ol**  **** | **186** | **Octan-3-one**  **** |
| **59** | **Caryophylene oxide ** | **81** | **P-cymene**  **** | **153** | **Menth-2-enol**  **** |
| **229** | **Alpha-terpinene ** | **216** | **Sabinene**  **** | **81** | **P-cymene**  **** |
| **Mentha Pulegium** | | | | | |
| **8** | **2-cyclohexen-1 ol ** | **154** | **Menthene**  **** | **156** | **Menthone**  **** |
| **18** | **3-acetyl-2.5-dimethylthiophene ** | **120** | **Glycocyanidine**  **** | **171** | **Neoisomenthol**  **** |
| **19** | **3-cyclopentylpropionic acid**  **** | **214** | **Rotundifolone ** | **127** | **Hexahydrofarnesyl acetone ** |
| **78** | **Cyclohexanol ** |  |  |  |  |
| **Llavandula Angustifolia** | | | | | |
| **53** | **Borneol ** | **216** | **Sabinene**  **** | **113** | **Geranyl acetate**  **** |
| **53** | **Camphor**  **** | **230** | **Terpineol**  **** | **144** | **Linalyl acetate**  **** |
| **59** | **Caryophylene oxide ** | **249** | **Alpha-bisabolol ** | **101** | **Farnesyl acetate ** |
| **Majorana Hortensis Moench** | | | | | |
| **52** | **Camphene**  **** | **191** | **P-Cymol**  **** | **172** | **Neophytadiene ** |
| **Rosmarinus Officinalis** | | | | | |
| **53** | **Borneol**  **** | **143** | **Linalol**  **** | **58** | **Caryophyllene**  **** |
| **52** | **Camphene** | **231** | **Terpinolen**  **** | **81** | **P-cymene**  **** |
| **53** | **Camphor** | **97** | **Eucalyptol**  **** |  |  |
| **Salvia officinalis** | | | | | |
| **48** | **Caffeic acid ** | **149** | **Luteolin ** | **207** | **Quercetin ** |
| **Marrubium Vulgare** | | | | | |
| **21** | **Acteoside ** | **22** | **Aesculin**  **** | **26** | **Alyssonoside ** |
| **48** | **Caffeic acid ** | **49** | **Caffeoylmalic acid ** | **55** | **Carnosol**  **** |
| **62** | **Chlorogenic acid ** | **72** | **P-coumaric acid ** | **82** | **Deacetylvitexilactone** |
| **89** | **4-hydroxybenzoic acid ** | **148** | **Lupeol**  **** | **204** | **Protocatechuic acid ** |
| **106** | **Ferulic acid ** | **151** | **Marrubenol**  **** | **214** | **Rosmarinic acid ** |
| **108** | **Forsythoside b ** | **152** | **Marrubic acid ** | **218** | **A sacranoside ** |
| **110** | **Gallic acid ** | **158** | **3-deoxo-15(s)-methoxyvelutine ** | **221** | **Samioside ** |
| **111** | **Gentisic acid ** | **187** | **Oleanolic acid ** | **222** | **Sinapic acid ** |
| **139** | **Leucosceptoside ** | **192** | **Peregrinin ** | **227** | **Syringic acid ** |
| **246** | **Vulgarin ** | **193** | **Peregrinol ** | **240** | **Umbelliferone**  **** |
|  |  | **247** | **Vulgarol**  **** | | |
| **Mentha Spicata** | | | | | |
| **5** | **1-isobenzofuranone ** | **92** | **Eriodictyol-7-o-glucoside ** | **125** | **Hesperidin ** |
| **64** | **Chrysosplenetin ** | **93** | **Erucic acid ** | **132** | **4-hydroxycoumarin ** |
| **86** | **Decuroside iii ** | **105** | **Ferreirin ** | **138** | **Kaempferol ** |
| **87** | **Demethylsulochrin ** | **118** | Ginkgolide   | **157** | **Meprednisone ** |
| **210** | **Rhamnocitrin ** | **219** | **Safingol ** | **208** | **Retusin ** |
|  |  |  |  | **224** | **Stearamide ** |
| **Teucrium Polium** | | | | | |
| **32** | **Aristolene ** | **116** | **Germacrene-d-4-ol ** | **213** | **Rosifoliol ** |
| **47** | **Cadinol ** | **129** | **Himachalene ** | **217** | **Sabinol**  **** |
| **54** | **Carene ** | **164** | **Alpha-muurolol ** | **232** | **Thujene** |
| **58** | **Caryophyllene** | **168** | **Myrtenol** | **233** | **Thymol** |
| **59** | **Caryophyllene oxide** | **194** | **Phellandrene** | **241** | **Valencene** |
| **77** | **Cubenol** | | | | |

| **Rosaceae** | | | | | |
| --- | --- | --- | --- | --- | --- |
| **Malus domestica** | | | | | |
| **62** | **Chlorogenic acid** | **197** | **Phloretin** | **207** | **Quercetin** |
| **72** | **P-coumaric acid** | **198** | **Phloridzin** | **211** | **Quercetin-3-rhamnoside** |
| **110** | **Gallic acid** | **203** | **Procyanidin** | | |
| **Rosa Damascena** | | | | | |
| **34** | **Astragalin** | **110** | **Gallic acid** | **161** | **Methyl gallate** |
| **60** | **Catechin** | **131** | **Phenethyl 3,4,5-trihydroxybenzoate** | **204** | **Protocatechuic acid** |
| **89** | **4-hydroxybenzoic acid** | **138** | **Kaempferol** | **207** | **Quercetin** |

| **Zingiberaceae** | | | | | |
| --- | --- | --- | --- | --- | --- |
| **Alpinia officinarum hance** | | | | | |
| **38** | **Bergamotol** | **123** | **Alpha-gurjunene** | **249** | **Alpha-bisabolol** |
| **42** | **Borneol** | **133** | **Isobutyl benzoate** | **251** | **Alpha-calacorene** |
| **43** | **Bornyl acetate** | **135** | **Isocaryophillene** | **163** | **Alpha-muurolene** |
| **44** | **Bornyl chloride** | **46** | **Butyric anhydride** | **173** | **Nerolidol** |
| **52** | **Camphene** | **53** | **Camphor** | **195** | **Phenethyl isobutyrate** |
| **76** | **Alpha-selinene** | **196** | **Phenylethyl isovalerate** | **237** | **Beta-farnesene** |
| **102** | **Fenchol** | **241** | **Valencene** | **248** | **Alpha-bergamotene** |
| **104** | **Fenchyl acetate** |  |  |  |  |
| **Curcuma longa** | | | | | |
| **11** | **2-methoxy-4-vinylphenol** | **137** | **Isolongifolol** | **2** | **Alpha-cedrene** |
| **33** | **Ar-turmerone** | **250** | **Alpha-cadinene** | | |
| **109** | **Furanodiene** | **115** | **Geranyl-p-cymene** |  |  |

| **Apiaceae** | | | | | |
| --- | --- | --- | --- | --- | --- |
| **Foeniculum vulgare** | | | | | |
| **94** | **Estragole** | **103** | **Fenchone** | **216** | **Sabinene** |
|  |  | **190** | **P-anisaldehyde** | | |
| **Ammi Visnaga** | | | | | |
| **7** | **1-tridecene** | **75** | **Croweacin** | **142** | **Limonene** |
| **13** | **2-methyl propyl butanoate** | **80** | **Cyclopentanol** | **145** | **Linalyl butyrate** |
| **17** | **3,4,5-trimethoxybenzaldehyde** | **100** | **Eugenol** | **146** | **Linalyl valerate** |
| **27** | **Amyl isobutyrate** | **101** | **Farnesyl acetate** | **155** | **Menthol** |
| **28** | **Anethole** | **104** | **Fenchyl acetate** | **212** | **Rose oxide** |
| **43** | **Bornyl acetate** | **113** | **Geranyl acetate** | **233** | **Thymol** |
| **70** | **Citronellyl isobutyrate** | **130** | **Humulene** | **134** | **Isobutyl valerate** |
| **71** | **Citronellyl propionate** |  |  |  |  |
| **Pimpinella Anisum** | | | | | |
| **12** |  | **91** | **Epoxypseudoisoeugen yl-2-methylbutyrate** | **205** | **Pseudoisoeugenyl-2- methyl butyrate** |
| **34** | **Anethole** | **117** | **Geyrene** | **37** | **Epoxypseudoisoeugen yl-2-methylbutyrate** |
| **79** | **Cycloisolongifolene** | **128** | **Hexatriacontane** | **160** | **Methyl eugenol** |
| **88** | **Docosane** | **177** | **Nonacosane** | | |

| **Asteraceae** | | | | | |
| --- | --- | --- | --- | --- | --- |
| **Artemisia Herba Alba** | | | | | |
| **10** | **2,5-bornanedione** | **53** | **Camphor** | **25** | **Alpha-thujone** |
| **23** | **Alhanin** | **63** | **Chrysanthenone** | **52** | **Camphene** |
|  |  | **114** | **Eucalyptol** | **229** | **Alpha-terpinene** |
| **Anacyclus Pyrethrum** | | | | | |
| **61** | **8-cedren-13-ol acetate** | **98** | **[alpha]-eudesmol acetate** | **220** | **Salvial-4(14)-en-1-one** |
| **163** | **Alpha-muurolene** | **236** | **Trans-isolongifolanone** | **245** | **Vetivenic acid** |
| **183** | **Occidentalol** |  |  |  |  |
| **Matricaria Camomilla** | | | | | |
| **29** | **Apigenin** | **62** | **Chlorogenic acid** | **207** | **Quercetin** |
| **30** | **Apigenin-7-o-glucoside** | **106** | **Ferulic acid** | **234** | **Trans-β-ionone** |
| **48** | **Caffeic acid** | **149** | **Luteolin** | | |

| **Lauraceae** | | | | | |
| --- | --- | --- | --- | --- | --- |
| **Cinnamomum Burmanni** | | | | | |
| **73** | **Coumarin** | **227** | **Syringic acid** | **138** | **Kaempferol** |
| **89** | **4-hydroxybenzoic acid** | **243** | **Vanillic acid** | **162** | **Methyl vanillate** |
| **107** | **Ficaprenol-10** | | | **223** | **Squalene** |
| **Laurus Nobilis** | | | | | |
| **40** | **Bicyclogermacrene** | **174** | **N-heptacosane** | **182** | **N-tricosane** |
| **54** | **Bornyl acetate** | **175** | **N-hexacosane** | **199** | **Phytol** |
| **52** | **Camphene** | **176** | **N-octacosane** | **223** | **Squalene** |
| **60** | **Catechin** | **180** | **N-pentacosane** | **181** | **N-tetracosane** |
| **170** | **N-docosane** |  |  | | |

| **Fabaceae** | | | | | |
| --- | --- | --- | --- | --- | --- |
| **Cassia Absus** | | | | | |
| **24** | **Alpha-linolenic acid** | **141** | **Lignoceric acid** | **188** | **Oleic acid** |
| **31** | **Arachidic acid** | **150** | **Margaric acid** | **189** | **Palmitic acid** |
| **35** | **Behenic acid** | **167** | **Myristic acid** | **225** | **Stearic acid** |
| **90** | **Eicosadienoic acid** | | | | |
| **Acacia Raddiana** | | | | | |
| **1** | **(e)-2-heptenal** | **68** | **Citronellol** | **178** | **Nonanal** |
| **2** | **(e)-2-octenal** | **69** | **Citronellyl acetate** | **179** | **Nonanoic acid** |
| **3** | **(e)-cinnamaldehyde** | **83** | **Decanal** | **201** | **Piperitone** |
| **9** | **2,4-nonadienal** | **84** | **Decanoic acid** | **202** | **P-menth-9-en-1-ol** |
| **14** | **2-nonanone** | **95** | **Ethyl 2, 4-decadienoate** | **235** | **Trans-carveol** |
| **15** | **2-octanone** | **96** | **Ethyl decanoate** | **238** | **Tridecane** |
| **16** | **2-phenyl ethyl benzoate** | **112** | **Geraniol** | **239** | **Tridecanol** |
| **20** | **Acetophenone** | **114** | **Geranyl acetone** | **36** | **Benzyl benzoate** |
| **45** | **Bulnesol** | **124** | **Heptanal** | **126** | **Hexadecane** |
| **50** | **Calamenene** | **136** | **Isoelemicin** | **57** | **Carvone** |
| **60** | **Catechin** | **159** | **Methyl cinnamate** | **160** | **Methyl eugenol** |
| **65** | **Cinnamic acid** | **173** | **Nerolidol** | | |
| **Glycyrrhiza Glabra** | | | | | |
| **4** | **(e,e)-farnesol** | **121** | **Glycyrrhetic acid** | **147** | **Liquiritin** |
| **85** | **Decanol** | **122** | **Glycyrrhizin** | **140** | **Licochalcone a** |
| **119** | **Glabridin** | | | | |

| **Myrtaceae** | | | | | |
| --- | --- | --- | --- | --- | --- |
| **Syzygium Aromaticum** | | | | | |
| **51** | Campesterol | **74** | Crategolic acid | **99** | Eugenin |
| **100** | Eugenol | **110** | **Gallic acid** | **138** | **Kaempferol** |
| **166** | Myricetin | **226** | Stigmasterol | **187** | **Oleanolic acid** |
| **244** | Vanillin | **39** | bicornin | **41** | Biflorin |
| **209** | Rhamnetin | | | | |
| **Myrtus Communis** | | | | | |
| **59** | Caryophyllene oxide | **112** | geraniol | **113** | Geranyl acetate |
| **143** | Linalool | **160** | methyl eugenol | **165** | myrcene |
| **169** | myrtenyl acetate | **230** | Terpineol | **57** | **Carvone** |
| **Eucalyptus Globulus** | | | | | |
| **52** | **Camphene** | **143** | **Linalol** | **66** | Citral |
| **67** | Citronellal | **113** | **Geranyl acetate** | **142** | **Limonene** |
|  |  | **228** | Tereticornate | | |

**Table 2**: Structures of phytoconstituents having exhibited the highest affinity towards the target proteins.

| Lig | Structure | Lig | Structure | Lig | Structure |
| --- | --- | --- | --- | --- | --- |
| L82 |  | L86 |  | L92 |  |
| L119 |  | L121 |  | L147 |  |
| L148 |  | L149 |  | L187 |  |
| L192 |  | L193 |  | L211 |  |
| L221 |  | L226 |  | L228 |  |

**
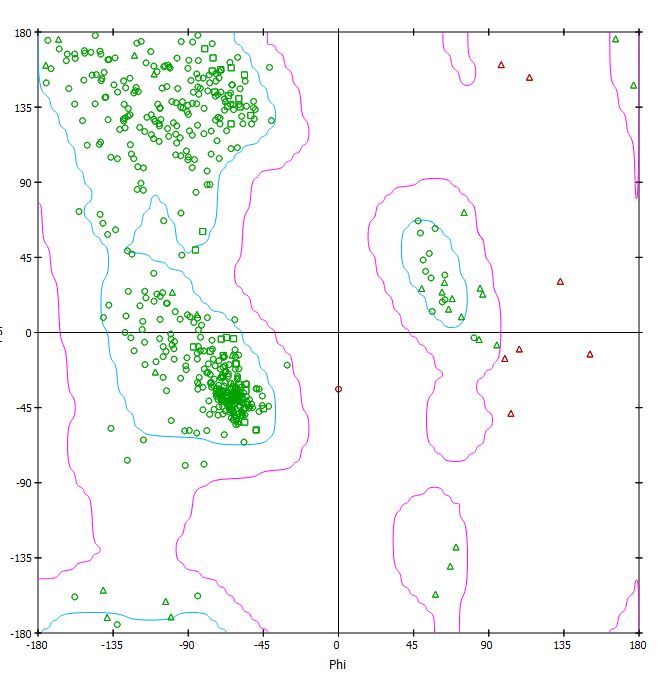
**

**Figure S1:** Ramachandran plot of BuChE.

**
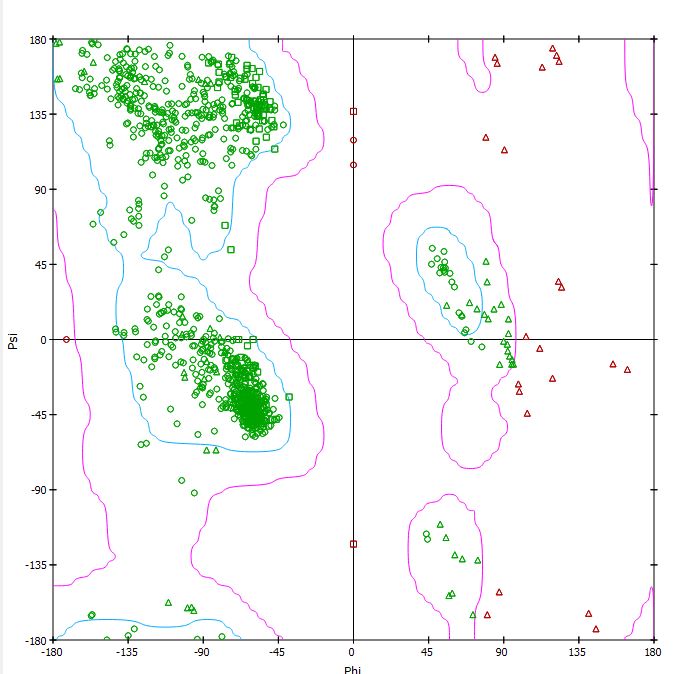
**

**Figure S1:** Ramachandran plot of AChE.
